# Supplementary material for: Adhesion to the Brain Endothelium Selects Breast Cancer Cells with Brain Metastasis Potential
Source: Int J Mol Sci. 2023 Apr 11;24(8):7087. doi: 10.3390/ijms24087087 (PMC10138870; doi:10.3390/ijms24087087)
Supplement: Supplementary file 1 [file ijms-24-07087-s001.zip › ijms-2334452-supplementary.pdf]

Adhesion to the Brain Endothelium Selects Breast Cancer Cells with Brain Metastasis Potential

Supplementary Figures:

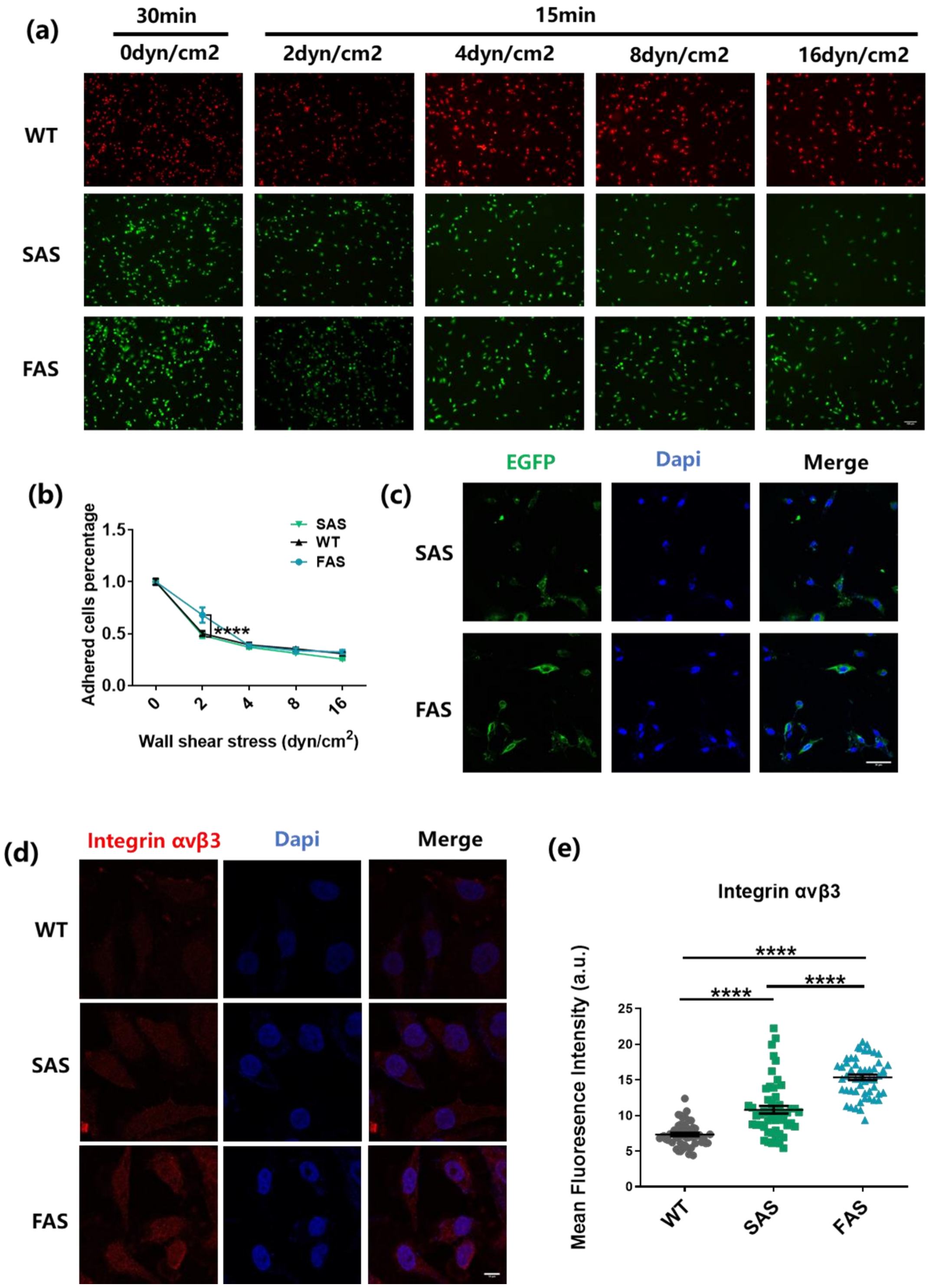

Figure S1. FAS cells showed enhanced adhesion strength. *(a, b) All groups were marked by cell*

*tracker and added into the flow chamber slides to adhere to the brain endothelium for 30 minutes. Then 2dyn/cm2, 4 dyn/cm2, 8 dyn/cm2, 16 dyn/cm2 shear flow were applied for 15 minutes in order. The cancer cells remaining on the brain endothelium after each application of shear flow were counted using 10x fluorescence microscope. Scale bar=100μm. n=3. (c) Representative images of the expression of EGFP in the SAS and FAS cells. These selected cells were free of the contamination of hCMEC/D6 cells. Scale bar=50μm. (d) The expression of integrin αvβ3 in all tumor cells were tested through immunofluorescence staining. Scale bar=10μm. (e) Quantification of the expressions of integrin αvβ3 in (d). The statistics among three groups were calculated based on one-way ANOVA with the post hoc Bonferroni test. n=50. The statistics were analyzed based on two-way ANOVA with post hoc Tukey test. All data were represented by mean ± SEM. (****p<0.0001)*

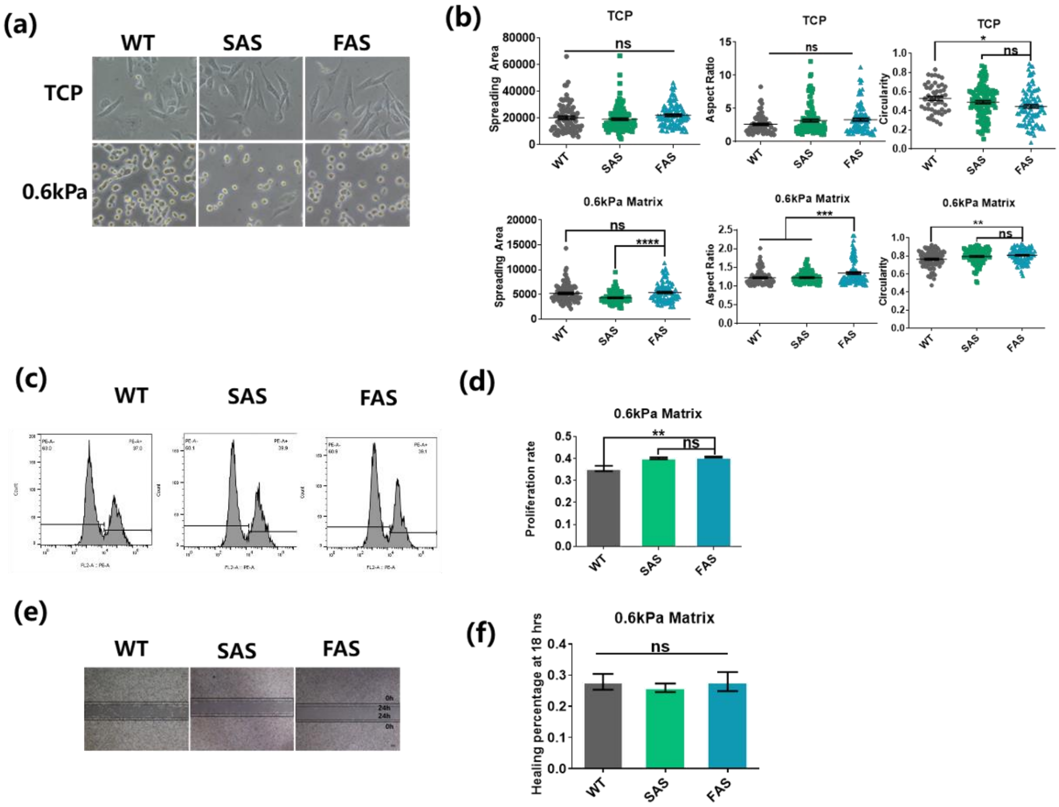

Figure S2. The selected breast cancer cells showed limited enhancement in adaptation to a soft brain environment. *(a,b) Morphology analysis shows that FAS cells adapt better on 0.6kPa soft matrices. All cells were seeded on 0.6kPa PA-gel or tissue culturing plastic overnight and 40x bright-field microscopic images were taken. ImageJ was used to analyze the spreading area and aspect ratio of cells. n=3; (c,d) FAS ceells have enhanced proliferation on 0.6kPa. All groups were seeded on 0.6kPa PA-gel overnight and then examined on proliferation rate using EdU kit. n=3; (e,f) All groups show no difference in migration ability on soft matrices. Cells were seeded on 0.6kPa PA-gel and a wound was generated using the insert. The healing rate at 24h was calculated using imageJ. Scale bar=100μm. n=3. The statistics were calculated using one-way ANOVA with post hoc Bonferroni test. All data were represented by mean ± SEM. (ns: no significance, *p<0.05, ** p < 0.01, *** p< 0.001, ****p<0.0001)*

Table S1. The list of all primers used in qPCR assays

**Gene Symbol**	**Front Primer**	**Reverse Primer**
ANGPTL4	TCCGTACCCTTCTCCACTTG	AGTACTGGCCGTTGAGGTTG
COX2	TTCAACACACTCTATCACTGGC	AGAAGCGTTTGCGGTACTCAT
EREG	CTGCCTGGGTTTCCATCTTCT	GCCATTCATGTCAGAGCTACACT
HBEGF	GGACCCATGTCTTCGGAAAT	CCCATGACACCTCTCTCCAT
ITGAV	CTCGGGACTCCTGCTACCTC	AAGAAACATCCGGGAAGACG
ITGB3	CCGTGACGAGATTGAGTCA	AGGATGGACTTTCCACTAGAA
ST6GALNAC5	CACTGGACGGATACCTCGGA	TCTGTCTGGTCAATCTGGGAG
PIEZO2	GACGGACACAACTTTGAGCCTG	CTGGCTTTGTTGGGCACTCATTG
SCNN1A	AGGGGAACAAGCGTGAGGA	GGTGGAACTCGATCAGGGC
LTBP1	CTTCCCCTGCCCGGTCT	CTGCATCTTTATAGTTCTCACCACCA
MUC1	TGCCGCCGAAAGAACTACG	TGGGGTACTCGCTCATAGGAT
GAPDH	GGAGCGAGATCCCTCCAAAAT	GGCTGTTGTCATACTTCTCATGG
CD44	CTGCCGCTTTGCAGGTGTA	CATTGTGGGCAAGGTGCTATT
ITGA4	CACAACACGCTGTTCGGCTA	CGATCCTGCATCTGTAAATCGC
ITGB1	CCTACTTCTGCACGATGTGATG	CCTTTGCTACGGTTGGTTACATT
ALCAM	TCCTGCCGTCTGCTCTTCT	TTCTGAGGTACGTCAAGTCGG
VCAM1	GGGAAGATGGTCGTGATCCTT	TCTGGGGTGGTCTCGATTTTA
ITGB2	TGCGTCCTCTCTCAGGAGTG	GGTCCATGATGTCGTCAGCC
ITGAL	TGCTTATCATCATCACGGATGG	CTCTCCTTGGTCTGAAAATGCT
SerpinB2	GTTCATGCAGCAGATCCAGA	CGCAGACTTCTCACCAAACA
